# Supplementary material for: What factors impact student performance in introductory physics?
Source: PLoS One. 2020 Dec 17;15(12):e0244146. doi: 10.1371/journal.pone.0244146 (PMC7746181; doi:10.1371/journal.pone.0244146)
Supplement: Attachment — Submitted filename: RefereeResponse_PLOSOne.docx [file pone.0244146.s001.docx]

*To the academic editor and reviewers,*

*We thank you for your time and consideration of our manuscript. The feedback you have provided will allow us to significantly strengthen the manuscript and make it more suitable for the PLOS One audience. We have addressed each of your concerns in-line below.*

*The formatting issues have been resolved and the ethics statement has been placed in the methods section.*

5. Review Comments to the Author

Please use the space provided to explain your answers to the questions above. You may also include additional comments for the author, including concerns about dual publication, research ethics, or publication ethics. (Please upload your review as an attachment if it exceeds 20,000 characters)

Reviewer #1: Reviewer comments for each prompt follow below each prompt.

1 and 2. Is the manuscript technically sound, and do the data support the conclusions? Has the statistical analysis been performed appropriately and rigorously?

The experiments, statistics, and other analyses are performed to a high technical standard and are described in sufficient detail for some of the work but not all. The techniques for collecting the data, identifying factors important to students’s experiences in introductory physics classes and arriving at the percentages in Table III are clearly described. The methods for evaluating the validity of conclusions that could be drawn from the differences in percentages are not provided. These methods seem critical for this data set that involves responses from a small number of participants (N=15 and 19).

*We have clarified: “As the number of participants in each category is small and this study is primarily qualitative, we do not conduct any inferential statistical tests. We draw conclusions based upon the fraction of students in each category who mentioned a particular code.”*

The abstract does not represent the findings accurately. The phrase ”such as collaborative interactions between students, were correlated with course performance” is misleading. The discussion of the data and the data analysis as presented do not adequately support drawing any conclusions on the effects on expected course performance. In particular, the abstract does not include a reference to one of the most significant looking influences on expected course performance. The data suggest that students who take physics more than a year after high school seem to perform worse. This difference in performance is as large or larger than any other effect considered in the analysis. The manuscript suggests that this effect may be due to the students being relatively uninterested in physics. Have the authors considered other possibilities such as students who “delay” forgetting more of the physics they learn.

*The reviewer makes a fair point that students who delay may simply have forgotten more physics, however prior research suggests that students’ conceptual understanding of physics is fairly stable over periods of approximately one year. Additionally, we have re-written the abstract to more accurately reflect the conclusions we were able to draw and the most important features of the results we collected.*

Echoing the above, it is not possible to tell what criteria the authors used to draw conclusions. If there were statistical methods applied, then they should be described.

*See above comment.*

3. No, according to the author's statement with the manuscript.

4. Is the manuscript presented in an intelligible fashion and written in standard English?

Yes except for a couple of points.

It would be helpful for Table III to include the N’s for the table headings “Better than predicted” and “Worse than predicted”.

*Done.*

It would be helpful for Table III to indicate which of the items had too few instances for the authors to include them in the quantitative analysis. It is confusing to see the percentages associated with them listed as the authors state in the text that they will not include them in the quantitative analysis.

*Done, and noted in the caption.*

Additional Comments:

The authors acknowledge that students may be reluctant to talk about their experiences in courses in which they feel they have performed poorly. The financial incentive seems like a neutral way to attract participants to the study. In my opinion, the more aggressive tactics to boost the numbers that included enlisting third parties (like diversity offices and department administrators) might feel threatening to the students. It would help if the manuscript included what the guidelines were for soliciting responses.

*We have added to the methods section: “*We note that no student was contacted more than four times over a period of 5 months to avoid harassing the students.*”*

Reviewer #2: The manuscript is of general interest in education. I am not sure the
conclusion meshes very well with the title of the paper though, because
the hidden variable are not quite and clearly identified as the ones
that affect the student performance. I do see lots of variables, but the
authors should summarize the "few" most important of all the listed
variables as the "Hidden variables that affect student performance," I
think.

*We have changed the title to “What factors impact student performance in introductory physics?”*

Other items:
1) The authors claim 34 students is a large number of students for such study. I am
not sure that 34 is enough for such lofty goals, but the study does involve
much research even with 34 students.

*For a qualitative study of this kind, 34 is indeed a large number of students to speak with. It is nearly 10% of the enrollment in the course.*

2) Line 30 should cite reference 6

*Done.*

There are a few typos, please check. Some found are on lines:245 (use 'they are'
rather than 'them'); 336 (use 'these'); line 353 (use 'neutral');
427 (use characteristics');

*Typos are fixed.*

3) Explain on the first apearance what the authors mean by N, see, for example
line 182. It's confusing when one first encounters it.

*We have defined N for the reader.*

Reviewer #3: This study tries to identify issues (hidden variables) that may be important for student experience and performance in introductory physics, especially for students weakly prepared for the course. Based on the interviews of 34 students, the authors identified these issues and categorized them into attitudes and practices. They then analyzed the difference between better-than-predicted and worse-than-predicted student groups in each of these issues. Some issues were found to have a negative or positive correlation with course performance: Out-of-class social interactions, extracurriculars, and athletics correlated with worse performance, while certain student characteristics (growth mindset and grit) correlated with better performance. Surprisingly, use of instructional resources showed no or a negative correlation with performance. In my opinion, this study does not reveal insightful hidden variables or provide practical guidelines specific to intro phys to improve student performance. Rather, it reaffirms that variables not specific to intro phys course such as personal attributes or pre-existing conditions (grit, leadership, mindset, gap year, extracurriculars, student athletes, etc.) are the strongest predictors of the course performance.

Major issues:

The interviews are retrospective. Students are more likely to be influenced by their experience in the courses that they are taking currently. It also appears that the 34 students took the intro phys course from different lecturers in different semesters. These variables will definitely confound the interpretation of their interviews.

*We apologize for not being clearer on this. All students took the same course in the same semester from the same lecturer. We have clarified this in the methods section. We have added a disclaimer as well: “*We also cannot rule out the possibility that students’ responses were skewed by other courses they were currently taking (e.g. contrasting their current and prior experiences).*”*

Overall, better and worse student groups identified most practices at a similar rate. Therefore, these practices might not be good predictors of student performance as the authors suggest. But it is also possible that underperforming students simply “do not know what they do not know” and are misled to think that they increased their knowledge by pursuing such practices.

*This is a good point, and we have added it to the appropriate section of the discussion.*

Since the most important attitudes identified from this study are growth mindset, grit, and confidence which are individual-related rather than course-related, students in the better-than-predicted group are more likely to be better in other courses. Have the authors compared average GPAs of the two groups based on other courses?

*This is a good point and we now discuss this in the discussion. Students in who perform better than predicted do have higher GPAs, but the difference is not significant. “*Finally, we note that all these attitudinal variables are individual-releated rather than course related, so one might expect that students who perform better than predicted in this course also perform better in other courses. We do indeed find that students who perform better than predicted have higher cumulative GPAs than students who perform worse than expected, but this difference is small and not significant (p = 0.13, Mann-Whitney U-test).*”*

The authors use the final exam score as a metric for performance while asking students questions not explicitly related to the final preparation. Student experience over the whole semester does not necessarily correlate with the final exam score, and I think this lack of correlation would be more pronounced among underperforming students.

*This is a fair point, and we now comment on it in the discussion when addressing limitations of the current study. We also point out that students did not mention radical shifts in their practices and attitudes during the semester in the interviews, so it seems likely that these practices were consistent across all exams and course assignments. “*A limitation of the current study is that we use final exam score as a measure of performance, but our questions to the students were about their experiences over the entire course and not just in preparation for the final exam. A more appropriate measure of course performance might therefore be final course grades, but we note that 40% of the final course grade is determined by the final exam, so there is a strong correlation. Furthermore, none of the students mentioned significant changes in the way that they approached or viewed the course during their interviews, so it seems likely that the behaviors they reported were stable over the course of the term.*”*

Minor questions:

It sounds like the one-hour companion course would be a great help for students. Did the authors find any positive effect of taking this course?

*There was an effect for some students. We have added to the results: “*Finally, we note that our prior statistical analyses [6] found no main effect of the companion course, but did find a positive effect for students with the lowest levels of prior preparation.*”*

One of the questions which I personally deem as most important did not make it to the list of issues: “Would you review solutions or re-work homework problems when studying?” Is it because most students did not mention this practice?

*We thank the reviewer for brining this point up, and we did indeed ask this as a follow-up question during the interview. We found that most students reported re-working the homework problems with the solutions nearby, which may not have been a helpful practice depending on how often students referenced the written solutions. We have added this to our definition of the codes and discussion of the results.*
